# Dual effect of the wheat ***Ph1*** locus on chromosome synapsis and crossover

**DOI:** 10.1007/s00412-017-0630-0

**Published:** 2017-04-01

**Authors:** Azahara C. Martín, María-Dolores Rey, Peter Shaw, Graham Moore

**Affiliations:** John Innes Centre, Norwich Research Park, Norwich, NR4 7UH UK

**Keywords:** Bouquet, Environment, Meiosis, Pairing, Telomeres, Wheat

## Abstract

Allopolyploids must possess a mechanism for facilitating synapsis and crossover (CO) between homologues, in preference to homoeologues (related chromosomes), to ensure successful meiosis. In hexaploid wheat, the *Ph1* locus has a major effect on the control of these processes. Studying a wheat mutant lacking *Ph1* provides an opportunity to explore the underlying mechanisms. Recently, it was proposed that *Ph1* stabilises wheat during meiosis, both by promoting homologue synapsis during early meiosis and preventing MLH1 sites on synapsed homoeologues from becoming COs later in meiosis. Here, we explore these two effects and demonstrate firstly that whether or not *Ph1* is present, synapsis between homoeologues does not take place during the telomere bouquet stage, with only homologous synapsis taking place during this stage. Furthermore, in wheat lacking *Ph1*, overall synapsis is delayed with respect to the telomere bouquet, with more synapsis occurring after the bouquet stage, when homoeologous synapsis is also possible. Secondly, we show that in the absence of *Ph1*, we can increase the number of MLH1 sites progressing to COs by altering environmental growing conditions; we show that higher nutrient levels in the soil or lower temperatures increase the level of both homologue and homoeologue COs. These observations suggest opportunities to improve the exploitation of the *Ph1* wheat mutant in breeding programmes.

**Electronic supplementary material:**

The online version of this article (doi:10.1007/s00412-017-0630-0) contains supplementary material, which is available to authorized users.

## Introduction

More than 70% of flowering plants are polyploids, most being allopolyploids. Allopolyploids such as wheat, canola, oats and cotton represent some of the world’s most important crops. Allopolyploids possess more than two complete sets of chromosomes, as a result of chromosome doubling (polyploidisation) following sexual hybridisation between related species possessing similar genomes. Production of viable gametes and subsequent fertility depends on the allopolyploid behaving as a diploid during meiosis, so that pairing, synapsis and recombination only take place between identical chromosomes (homologues) rather than between related chromosomes (homoeologues). The formation of at least one crossover (CO) link between homologous pairs (the obligate CO) is necessary for accurate segregation and balanced gametes (Zickler and Kleckner 1999). The high levels of allopolyploidy suggest that upon polyploidisation, plants may already have had mechanisms in place for meiotic sorting of homologues from homoeologues, providing the new allopolyploid with some fertility until improved by modification and stabilisation of the meiotic process.

Bread wheat (*Triticum aestivum*) is a hexaploid species possessing three related (homoeologous) ancestral genomes (termed AABBDD), derived from three different diploid species. Each genome set consists of seven chromosomes (1A–7A, 1B–7B and 1D–7D; 6*x* = 2*n* = 42), possessing highly similar gene order and content. Despite similarity between homoeologues, wheat behaves as a diploid during meiosis, with every chromosome synapsing and recombining only with its true homologue. This phenotypic behaviour is predominantly controlled by *Ph1*, a dominant locus on chromosome 5B (Riley and Chapman 1958; Sears and Okomoto 1958). The *Ph1* locus most likely arose during wheat polyploidisation, rather than being already present in one of the original wheat ancestral diploids (Chapman and Riley 1970). The *Ph1* locus was originally discovered through scoring the metaphase I phenotype of hexaploid wheat hybrids lacking the whole 5B chromosome (Riley and Chapman 1958; Sears and Okomoto 1958) and is therefore defined as a deletion mutant phenotype in wheat and its hybrids, controlling correct pairing (Holm and Wang 1988; Prieto et al. 2004) and suppressing homoeologous recombination (Dhaliwal et al. 1977). However, the detailed mode of action of the *Ph1* locus on these processes remains unclear.

Sexual hybridisation between wheat and rye produces an interspecific hybrid containing haploid sets of wheat and rye chromosomes, which can be exploited in breeding to introgress rye chromosome segments carrying agronomic traits into wheat. However, introgression is prevented by the presence of *Ph1*, which suppresses COs between homoeologues (mean < 1 CO per meiocyte at metaphase I (Martín et al. 2014)). In *Ph1* deleted wheat-rye hybrids, COs are increased (mean 7.8) (Martín et al. 2014), reflecting the loss of the suppressing effect of *Ph1* on CO formation between homoeologues. A single *Ph1* deletion mutant, developed in hexaploid wheat variety Chinese Spring (CS) (CS *ph1b*, generated by Sears in 1977 (Sears 1977)), has been used worldwide in breeding programmes to introgress wild relative chromosome segments into wheat. Further deletion mutants of the *Ph1* locus have also been subsequently developed and exploited (Roberts et al. 1999; Al-Kaff et al. 2008; King et al. 2016).

It has recently been proposed that *Ph1* has a dual effect during meiosis (Martín et al. 2014). The first effect takes place early in meiosis, where *Ph1* promotes homologous synapsis, rather than suppress homoeologous synapsis. In hexaploid wheat, ring bivalents, with occasional rod bivalents, are observed at metaphase I in all meiocytes when *Ph1* is present, consistent with regular homologue pairing (Martín et al. 2014). In the CS *ph1b* (*Ph1* lacking) mutant, ring bivalents, with a few more rod bivalents, are still observed at metaphase I in close to 50% of meiocytes, reflecting regular homologous pairing. In the remaining meiocytes however, a variable level of multivalents and univalents is observed, involving up to 8 of the 42 chromosomes (Martín et al. 2014). Other *Ph1* deletion mutants have also shown a lack of multivalents at metaphase I in variable numbers of meiocytes (Roberts et al. 1999). Thus, although the loss of *Ph1* allows some homoeologues to crossover in hexaploid wheat, the fact that there are no multivalents in every single meiocyte suggests that homoeologous synapsis is not the main effect of *Ph1*. Consistent with this, in the wheat-rye hybrid, a similar level of overall homoeologous synapsis is achieved by pachytene whether or not *Ph1* is present, indicating that homoeologous synapsis is independent of *Ph1* (Martín et al. 2014). This led to the suggestion that *Ph1* may promote homologous synapsis rather than prevent homoeologous synapsis. However, it remained uncertain how homoeologous synapsis was suppressed in hexaploid wheat.

The second effect of *Ph1* occurs later in meiosis, where it affects the level of CO formation.

Immunolocalisation analysis, using the DNA mismatch repair protein MLH1, revealed that *Ph1* affects progression of MLH1 sites to COs (Martín et al. 2014). MLH1 is part of the main class I interfering CO pathway in plants (Mezard et al. 2007) and is required for the resolution of double Holliday junctions as COs. In most species, all MLH1 sites on synapsed chromosomes become COs (Ashley et al. 2001; Lhuissier et al. 2007). In wheat-rye hybrids, similar numbers of MLH1 sites are detected whether or not *Ph1* is present (Martín et al. 2014). However, the presence of *Ph1* prevents progression of virtually all MLH1 sites to COs, preventing recombination. Conversely, the absence of *Ph1* allows progression of one third of MLH1 sites to COs (Martín et al. 2014), suggesting a role for *Ph1* in the regulation of homoeologue MLH1 site resolution. In hexaploid wheat itself, independent of *Ph1*, similar numbers of MLH1 sites are also observed on synapsed chromosomes at diplotene (Martín et al. 2014); however, the level of COs is only consistent with the number of MLH1 sites when *Ph1* is present. Thus, in *Ph1-*deficient wheat and its hybrids, CO level is lower than predicted*.* It is unclear which other factors are involved in the regulation of homoeologous COs in the absence of *Ph1*, but it would be important to assess whether the level of COs can be increased further to correspond to the number of MLH1 sites observed.

In the present study, we aim to elucidate the two effects of *Ph1* during meiosis. Firstly, we investigate how homoeologous synapsis is suppressed in hexaploid wheat, facilitating homologous synapsis. We also explore the timing of homoeologous synapsis in relation to the telomere bouquet and the progression of homologous and homoeologous synapsis in wheat lacking *Ph1*. Secondly, we assess whether it is possible to increase the number of MLH1 sites becoming COs. To do that, we investigate the effect of selected environmental factors on the progression of MLH1 into COs in the absence of *Ph1*, in both wheat and wheat-rye hybrids.

## Materials and methods

### Plant material

Anthers were harvested from hexaploid wheat *Triticum aestivum* cv. Chinese Spring (CS) (*6x* = 2*n* = 42; AABBDD) and from wheat-rye hybrids—crosses between rye *Secale cereale* cv. Petkus (2*x* = 2*n* = 14; RR) and hexaploid wheat (CS)—in both cases either carrying or lacking the *Ph1* locus (*ph1b* deletion) (Sears 1977).

For meiotic studies, the seedlings were vernalised for 3 weeks at 8 °C and then transferred to a controlled environmental room until meiosis with the following growth conditions: 16/8 h, light/dark photoperiod at 20 °C day and 15 °C night, with 70% humidity. For the experiments on temperature, the growth conditions were 16/8 h, light/dark photoperiod at either 13 or 30 °C constantly, with 70% humidity. Tillers were harvested when the flag leaf was starting to emerge, and anthers collected. For each dissected floret, one of the three synchronised anthers was squashed in 0.1% acetocarmine stain and examined under the light microscope to identify the stage of meiosis. The two remaining anthers were used for the various studies.

### Genome in situ hybridisation during meiotic metaphase I

Anthers were collected as described in the “Plant material” section. Chromosome preparation, probe preparation and GISH were carried out as described previously (Cabrera et al. 2002).

### Immunolocalisation of meiotic proteins

Meiocytes of wheat and wheat-rye hybrids at selected stages of meiosis were embedded in acrylamide pads to preserve their 3D architecture (Martín et al. 2014), and immunolocalisation of meiotic proteins ASY1 and ZYP1 was performed as described previously (Martín et al. 2014). The following antibodies were used in this paper: Anti-TaASY1 (Boden et al. 2009) raised in rabbit and used at a dilution of 1:250; anti-HvZYP1 (Colas et al. 2016) raised in rat and used at a dilution of 1:200. The polyacrylamide pads were mounted in Prolong Diamond antifade reagent (Thermo Fisher Scientific Molecular Probes, Eugene, OR, USA) and left to cure for 2 or 3 days (in order to reach an optimum 1.47 refractive index) before being sealed with nail varnish. Images were collected as described in the “Image acquisition and analysis” section. Since antibodies do not always tolerate the aggressive procedures carried out during fluorescence in situ hybridisation (FISH), to detect meiotic proteins by immunofluorescence together with DNA probes by FISH, the *x*-*y* position of each collected image was automatically saved. In this way, it was possible to reprobe the slides subsequently with FISH and return to the same nuclei to collect further images.

### Fluorescence in situ hybridisation during meiotic prophase

When simultaneous immunolocalisation and FISH was not required, meiocytes were fixed in 3:1 absolute ethanol/glacial acetic acid (*v*/*v*) for 20 min, rinsed twice in 1× PBS and macerated with a pestle to release the meiocytes. Fifteen microlitres of meiocyte suspension was transferred onto a poly-L-lysine-coated slide and left to air dry for a few minutes. FISH was carried out as described previously (Cabrera et al. 2002) with some modifications: No pepsin treatment was performed in order to maintain the structure of the nuclei, and no dehydration steps were carried out. Telomere repeat sequence (TRS) probe was amplified by PCR as described previously (Cox et al. 1993) and labelled with tetramethyl-rhodamine-5-dUTP (Roche Applied Science, Mannheim, Germany) by nick translation as described previously (Cabrera et al. 2002). The repetitive sequence Spelt52 was amplified by PCR as described previously (Salina et al. 2004) and labelled with DIG-11-dUTP using the DIG-nick translation mix (Roche Applied Science, Mannheim, Germany). The repetitive sequence 4P6 (Zhang et al. 2004) was amplified by PCR using the primers designed for this study 5′TGCATTTTCCTACAAGTAATTGG3′ and 5′TGTCTCAGTTAACACTGCGC3′, which produce a band of 507 bp. 4P6 was labelled using the DIG-nick translation mix (Roche Applied Science, Mannheim, Germany) according to the manufacturer’s instructions. Digoxigenin-labelled probes were detected with anti-digoxigenin-fluorescein Fab fragments (Roche Applied Science, Mannheim, Germany).

When simultaneous immunolocalisation and FISH were needed, immunolocalisation was first performed as described in the “Immunolocalisation of meiotic proteins” section. Once the images had been collected and the *x*-*y* positions saved, the positions on the slides of the coverslips with the attached polyacrylamide pads were marked with a diamond slide-marking pen in order to be able to replace them in exactly the same position after the FISH labelling. The coverslips with the attached pads were carefully removed from the slides, washed in washing buffer (1× PBS + 0.1% Tween + 1 mM EDTA) (4 × 15 min) and incubated overnight in the same buffer. The pads were fixed in 4% formaldehyde (freshly made from paraformaldehyde) for 30 min and washed in 2× SSC (3 × 15 min). DNA probes and hybridisation mix were prepared as described in this section. The coverslips with attached pads were placed on slides, and 80 μl of denatured hybridisation mix was added to each coverslip and left to infiltrate the pads for 30 min. The slides were denatured for 7 min at 75 °C in a ThermoBrite Slide Processing System (Leica Biosystems, Wetzlar, Germany), and hybridisation was carried out in a humid chamber at 37 °C overnight. Post-hybridisation washes were as described previously (Cabrera et al. 2002), but using double the washing times. After incubation with the appropriate second antibody for 1 h at room temperature and then at 4 °C overnight, pads were counterstained with DAPI (1 μg/ml). The coverslips with attached pads were finally replaced on their original slides in exactly the same position (using the diamond pen alignment marks) and mounted in Prolong Diamond. Images were then collected using the stored *x*-*y* positions for each nucleus.

### Nutrient solution treatment

After vernalisation as described in the “Plant material” section, seedlings were sown in 500 g of John Innes Cereal Mix compost placed in cylindrical plastic pots 14 cm in diameter and 12 cm high. The John Innes Cereal Mix used in this study was produced by W E Hewitt & Son Ltd., 45 Cambridge Road, Cosby, Leicester, LE9 ISJ, UK, that describes its composition as follows: 40% peat, fine sphagnum peat from central Ireland; 40% sterilised soil; 20% washed horticultural grit 1–5 mm; 1.3 kg/m^3^ PG mix base fertiliser 14:16:18 + TE; 1 kg/m^3^ Osmocote®; wetting agent; 3 kg/m^3^ Maglime; and 300 g/m^3^ Exemptor®.

Five replicates were used for wheat lacking the *Ph1* locus; 15 replicates were used for wheat-rye hybrids lacking *Ph1*, and five plants were used for wheat-rye hybrids carrying *Ph1*. All plants were irrigated once a week with 100 ml of a nutrient solution (a modification of the Hoagland’s solution (Hoagland and Arnon 1950)) at the stem elongation stage of the vegetative stage (Zadoks growth stage 31 (Zadoks et al. 1974; Tottman 1987)), 2 to 3 weeks before meiotic metaphase I. The composition of the nutrient solution was KNO_3_ (12 mM), Ca(NO_3_)2·4H_2_O (4 mM), NH_4_H_2_PO_4_ (2 mM), MgSO_4_·7H_2_O (1 mM), NaFe-EDTA (60 mM), KCl (50 μM), H_3_BO_3_ (25 μM), MnSO_4_·H_2_O (2 μM), ZnSO_4_ (4 μM), CuSO_4_·5H_2_O (0.5 μM) and H_2_MoO_4_ (0.5 μM). After irrigating (minimum twice), anthers were collected and staged as described in the “Plant material” section, fixed in freshly prepared 3:1 absolute ethanol/glacial acetic acid (*v/v*) for at least one week and stained by the conventional Feulgen technique.

### Temperature treatment

For both the low and high temperature treatments, five replicates of wheat and five replicates of wheat-rye hybrids, both lacking *Ph1*, were used.

For the low temperature treatment, plants were transferred to a controlled environment room at 13 °C during the stem elongation stage of the vegetative stage when the flag leaf is just visible but still rolled (between Zadoks growth stage 37 and stage 39 (Zadoks et al. 1974; Tottman 1987)), 7 days before meiotic metaphase I.

For the high temperature treatment, plants were transferred to a controlled environment room at 30 °C at the early boot stage of the vegetative stage when the flag leaf sheath is extending (Zadoks growth stage 41 (Zadoks et al. 1974; Tottman 1987)), 2 days before meiotic metaphase I.

Since the length of meiosis is shorter at higher temperatures than at lower temperatures, the length of the high and low treatments was varied to ensure completion of the meiotic process at each treatment temperature. Thus at 30 °C, 2 days were more than sufficient to ensure completion of the meiotic process, while minimising the time available for adverse effects of high temperature on the CS plant. Conversely, at 13 °C, 7 days were required to ensure that meiosis could complete, because of the delaying effect of the cold temperature.

### Image acquisition and analysis

Meiotic metaphase I nuclei stained with acetocarmine or Feulgen were imaged using a Leica DM2000 microscope equipped with a Leica DFC450 camera and controlled by LAS v4.4 system software (Leica Biosystems, Wetzlar, Germany). Images were processed using Adobe Photoshop CS5 (Adobe Systems Incorporated, USa) extended version 12.0 × 64.

Meiotic metaphase I nuclei labelled by GISH were imaged using a Leica DM5500B microscope equipped with a Hamamatsu ORCA-FLASH4.0 camera and controlled by Leica LAS X software v2.0. Images were processed using Fiji (an implementation of ImageJ, a public domain program by W. Rasband available from http://rsb.info.nih.gov/ij/).

Polyacrylamide-embedded meiocytes were optically sectioned using a Leica TCS SP5II confocal laser scanning microscope (CLSM) controlled by Leica LAS-AF v.2.7 software. Z-stacks were deconvolved using Huygens Essential (Scientific Volume Imaging BV). Projections and analysis of 3D pictures were performed using Fiji.

### Statistical analyses

Statistical analyses were performed using STATISTIX 10.0 software (Analytical Software, Tallahassee, FL, USA). Analysis of variance (ANOVA) was based on randomised blocks. These included in wheat, cosine (univalents in Hoagland treatment), square root (ring bivalents at 13 °C) and logarithmic (rod bivalents at 30 °C) transformations; in wheat-rye hybrids in the absence of the *Ph1* locus, square (univalents in Hoagland treatment), cosine (rod bivalents in Hoagland treatment and univalents at 13 °C) and logarithmic (chiasmata/COs in Hoagland treatment) transformations; and in wheat-rye hybrids in the presence of the *Ph1* locus, cosine (univalents, rod bivalents and chiasmata/COs in Hoagland treatment) transformations. Means were separated using the least significant difference (LSD) test with a probability level of 0.05.

## Results and discussion

### Homoeologous synapsis is suppressed during the telomere bouquet stage independently of *Ph1*

It has been suggested that the *Ph1* locus promotes homologous synapsis rather than suppressing homoeologous synapsis. However, the mechanism through which this is achieved is unknown. In most species, including hexaploid wheat, homologues gradually align and synapse during meiosis, while telomeres cluster forming a bouquet at one pole of the nucleus (Zickler and Kleckner 1999; Prieto et al. 2004; Griffiths et al. 2006; Zhang et al. 2014; Klutstein and Cooper 2014). In yeast, it has been proposed that the telomere bouquet can restrict ectopic pairing between homologous sites in non-homologous chromosomes and so promotes homologous synapsis (Niwa et al. 2000; Davis and Smith 2006). If homoeologous pairing is also restricted in hexaploid wheat during the telomere bouquet, this would provide an explanation of how regular bivalent formation between homologues at metaphase I is achieved in wild-type wheat. Given the number and size of wheat chromosomes in hexaploid wheat, it would be very difficult to follow the relative proportion of overall homologous versus homoeologous synapsis during the telomere bouquet stage. However, the use of wheat-rye hybrids facilitates this study. Wheat-rye hybrids possess only homoeologous chromosomes; no homologues are present. Therefore, only homoeologous synapsis can occur. We have previously shown that in these hybrids, homoeologous synapsis is observed at late zygotene and pachytene, after dispersal of the telomere bouquet (Martín et al. 2014). However, it was unclear whether this homoeologous synapsis occurred at the telomere bouquet stage or only later.

**Fig. 1:**
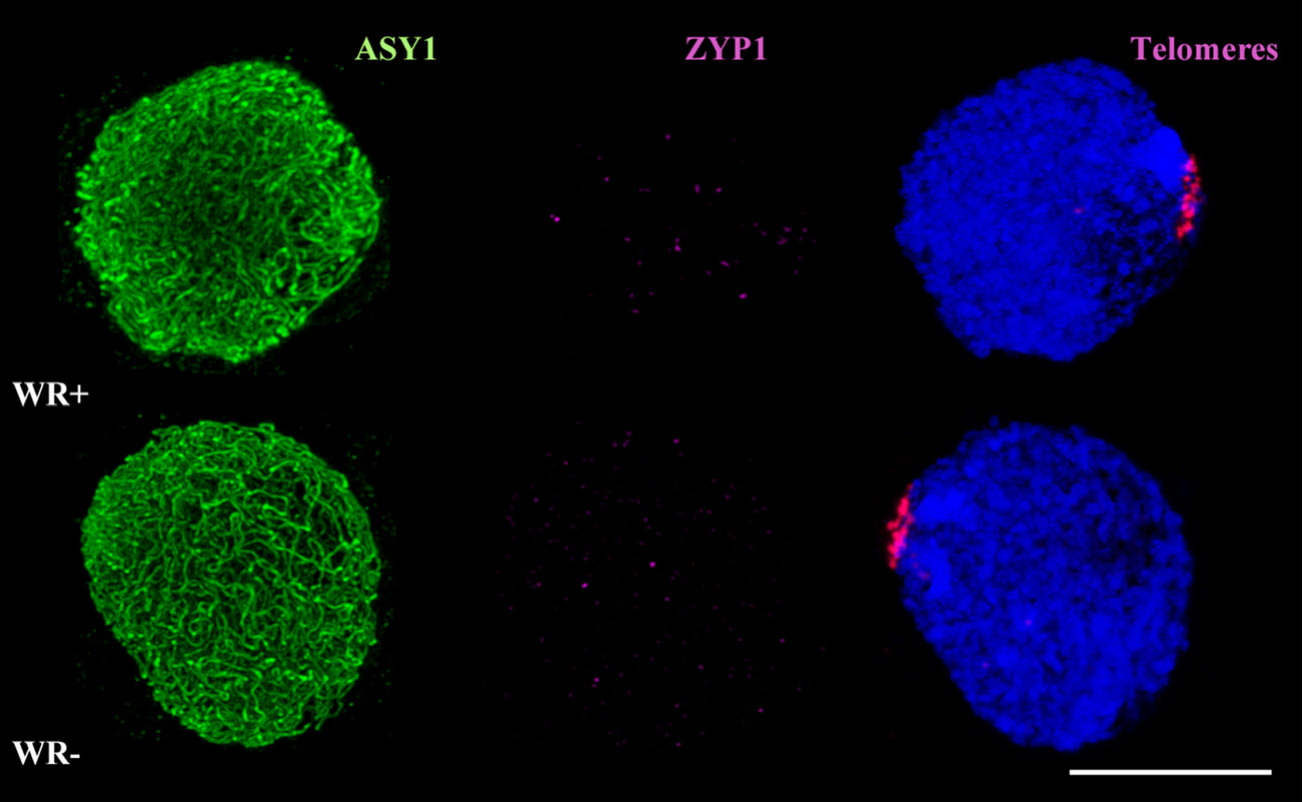
Homoeologous synapsis in wheat-rye meiocytes in the presence (WR+) and absence (WR−) of the *Ph1* locus during the telomere bouquet. Immunolocalisation of meiotic proteins ASY1 (*green*) and ZYP1 (*magenta*) combined with telomeres (*magenta*) labelled by FISH. Little or no ZYP1 labelling was detected in wheat-rye meiocytes with or without *Ph1* during the telomere bouquet stage (group 1), indicating little synapsis between homoeologues. Thus, homoeologous synapsis occurs mostly after the telomere bouquet stage. DAPI staining in blue. *Scale bar* represents 10 μm

Here, we used wheat-rye hybrids to investigate the timing of homoeologous synapsis in relation to the telomere bouquet. We combined FISH, to label the telomeres, with immunolocalisation of the meiotic proteins ASY1 and ZYP1. ASY1 is part of the lateral elements of the synaptonemal complex and is loaded before synapsis. ZYP1 is part of the central element of the synaptonemal complex and is present only where chromosomes are synapsed. For the purpose of this study, we classified the hybrid meiocytes into three groups, based on the number of telomeres detected: 1 to 5 telomere groups, defining the telomere bouquet stage (group 1); 6 to 14 telomere groups, identifying early bouquet dispersal (group 2); and more than 14 telomere groups, identifying when the telomere bouquet has dispersed (group 3). FISH and immunolocalisation analysis show that there are no long tracks of ZYP1 in wheat-rye hybrid meiocytes during the telomere bouquet, whether *Ph1* is present or absent (Fig. 1). The little ZYP1 that is present in some of the cells during the telomere bouquet stage is shown by very short tracks of ZYP1 (Online Resource 1), which may indicate an attempt to start the synapsis process, although the polymerisation process fails to progress further. In the presence of *Ph1*, 87% of meiocytes show these short tracks of ZYP1, with no ZYP1 present in the remaining meiocytes (Online Resource 1). Conversely, when *Ph1* is absent*,* only 17% of meiocytes show short tracks of ZYP1 (Online Resource 1), suggesting slight delay compared to when *Ph1* is present. In any case, long tracks of ZYP1 are only observed after the telomere bouquet stage, whether or not *Ph1* is present. Homoeologous synapsis thus mostly occurs after the telomere bouquet has dispersed (Online Resource 2), and not during the telomere bouquet stage as happens with homologous synapsis.

### Homologous synapsis is delayed in the absence of *Ph1*

If suppression of homoeologous synapsis during the telomere bouquet is independent of the presence of *Ph1*, we would expect to observe regular bivalent formation at metaphase I in 100% of meiocytes, independently of the presence of *Ph1*. However, some multivalents are observed in wheat in the absence of *Ph1* (Martín et al. 2014), implying that there is at least some degree of synapsis between homoeologues. Even so, in the absence of *Ph1*, there are multivalents in less than 50% of meiocytes; and even when multivalents are present, only up to 8 of the 42 chromosomes are involved (Martín et al. 2014). More than half of the meiocytes show regular bivalent configurations, indicating that homoeologous synapsis is mainly prevented in wheat even in the absence of *Ph1*, consistent with suppression of homoeologous synapsis at the telomere bouquet stage. This suggests, as reported, that *Ph1* may promote homologous synapsis rather than preventing homoeologous synapsis, and in the absence of *Ph1*, disruption of this process may allow a degree of homoeologous synapsis to occur.

**Fig. 2:**
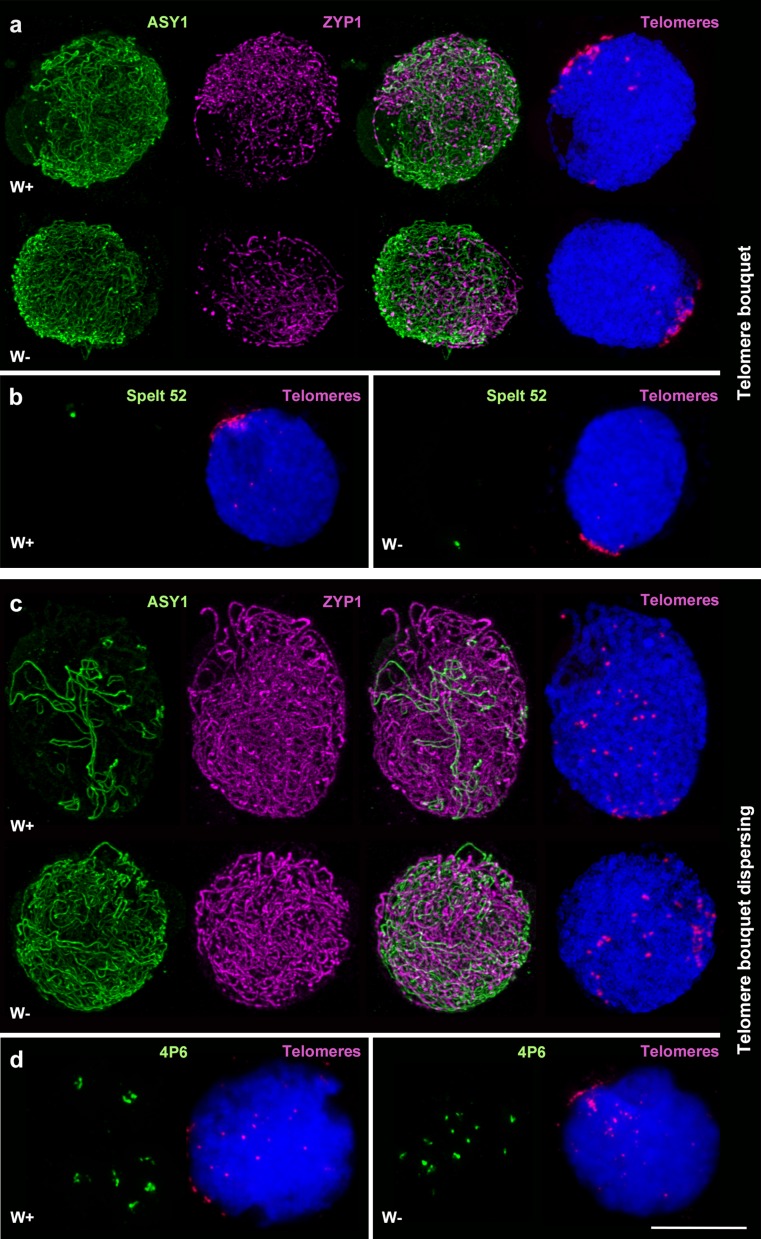
Progression of pairing and synapsis in wheat in the presence (W+) and absence (W−) of the *Ph1* locus. **a**, **c** Immunolocalisation of meiotic proteins ASY1 (*green*) and ZYP1 (*magenta*) combined with telomeres (*magenta*) labelled by FISH in W+ and W− meiocytes. **a** During early telomere bouquet dispersal (group 2), levels of synapsis were similar in both W+ and W−. **c** When the telomere bouquet is dispersed but ASY1 and ZYP1 do not colocalise yet (group 3), levels of synapsis were higher in W+ revealing a delay in synapsis in W−. **b** FISH using Spelt 52 (*green*) and a telomere (*magenta*) probe during the telomere bouquet (group 1). Spelt 52 produce a single signal in the distal region of chromosome 4BS. Only one signal was observed in both W+ and W− during the telomere bouquet confirming the data shown in **a**. **d** FISH using 4P6 (*green*) and a telomere (*magenta*) probe during telomere bouquet dispersal (group 2). 4P6 labels seven interstitial sites on chromosomes of the D genome. In the figure, only 7 signals are observed in W+ during the telomere bouquet dispersal, while 12 signals are shown in W−. FISH results reveal a delay in pairing in the absence of *Ph1*, confirming the delayed synapsis shown in **c**. DAPI staining in *blue*. *Scale bar* represents 10 μm

In order to assess the dynamics of overall chromosome synapsis in wheat and to identify any difference in the timing of synapsis in the absence of *Ph1*, we combined telomere labelling and immunolocalisation of ASY1 and ZYP1 proteins. Meiocytes from wheat, in the presence and absence of *Ph1*, were analysed from the beginning of telomere bouquet formation until the telomeres had completely dispersed. For the purpose of this study, we again divided the meiocytes into three groups, based on the number of telomere groups detected: 1 to 5, defining the telomere bouquet stage (group 1); 6 to 19, identifying early bouquet dispersal (group 2); and 20 or more telomere groups with no ASY1-ZYP1 colocalisation, identifying when the telomere bouquet is dispersed but total synapsis is not yet completed (group 3). Meiocytes with colocalisation of ASY1 and ZYP1 (occurring in wheat after pachytene) were not considered further in the analysis. Analysis suggests that the presence or absence of *Ph1* had no effect on the level of synapsis at the telomere bouquet stage (group 1) or during early bouquet dispersal (group 2) (Fig. 2a). However, when telomeres were dispersed (group 3), the level of ASY1 labelling was higher in the absence of *Ph1*, indicating a lower level of synapsis at that stage (Fig. 2c). By the end of pachytene, most chromosomes were synapsed in the absence of *Ph1* (Online Resource 3), indicating eventual completion of synapsis in most meiocytes even when *Ph1* was absent. These observations suggest that even if synapsis completes whether or not *Ph1* is present, progression of synapsis is slower in the absence of *Ph1* (as shown in group 3) with respect to the telomere dynamics.

In order to assess whether pairing was actually delayed in groups 1 and 2, FISH analysis was performed with specific probes to distal sites close to the telomere regions and to more proximal sites (see “Materials and methods” section). Probe Spelt52 produces a single signal in the distal region of 4BS chromosomes. One signal was observed at the telomere bouquet stage (group 1), whether *Ph1* was present or absent, indicating that only homologues associate at the telomere bouquet stage, independent of *Ph1* (Griffiths et al. 2006) (Fig. 2b). To study the pairing of more proximal chromosome sites, we used the specific probe 4P6, which labels seven interstitial sites on the D genome (Zhang et al. 2004) and which can be used to follow the dynamics of more interstitial sites undergoing later pairing. Meiocytes in which pairing has not occurred will exhibit 14 signals, 7 from each homologue, while meiocytes that have undergone complete regular pairing will exhibit only 7 signals. Therefore, meiocytes with either unpaired or incorrectly paired sites will exhibit between 8 and 14 signals (the probe cannot distinguish between unpaired and incorrectly paired sites). Here, meiocytes in the presence and absence of *Ph1* were collected at the stage of telomere bouquet dispersal (group 2, with 6 to 19 telomere groups detected), to identify any delay in pairing. In the presence of *Ph1*, most of the seven pairs of sites were associated, with 85.7% of them displaying 7 to 10 signals (Fig. 2d; Online Resource 4). In contrast, in the absence of *Ph1*, only 25% of the meiocytes displayed 7 to 10 signals, the remainder still showing more than 10 signals (Fig. 2d; Online Resource 4). This difference is consistent with a delay in pairing. It has been previously reported that the length of meiotic prophase I is similar whether or not *Ph1* is present (Bennett et al. 1971, 1974). Therefore, it is unlikely that the differences in pairing or synapsis related to the absence of *Ph1* are due to a gross difference in the timing of meiotic stages.

Thus, both synapsis and FISH analysis suggest that in the absence of *Ph1*, homologous chromosomes recognise each other during the telomere bouquet, but synapsis progresses more slowly. Since synapsis between homoeologues only occurs after the telomere bouquet, the delay of homologous synapsis in the absence of *Ph1* provides an opportunity for homoeologues to synapse after telomere bouquet dispersal. Previous yeast meiotic studies show that delayed pre-meiotic replication can subsequently delay chromosome pairing (Borde et al. 2000; Murakami et al. 2003). We have also previously reported altered pre-meiotic replication and chromatin in the absence of *Ph1* (Greer et al. 2012), proposing a possible subsequent effect on chromosome pairing and synapsis. Thus, this may explain the delayed synapsis found in the absence of *Ph1*.

### The level of CO formation in wheat and wheat-rye hybrids in the absence of *Ph1* is affected by the nutrient composition in the soil

The above data provide an explanation for how homoeologous pairing and synapsis might occur in hexaploid wheat in the absence of *Ph1*. However, it does not tell us anything about the effect of *Ph1* on CO formation. Fortuitously, our wheat-rye hybrids were grown recently, with and without *Ph1*, for chromosome doubling experiments at the Institute for Sustainable Agriculture (CSIC), Córdoba, Spain. Plants were grown outdoors in pots and treated with a modified Hoagland solution during their growth, in order to produce healthier plants for chromosome doubling. Surprisingly, when analysing the metaphase I configurations, we observed a higher level of chiasmata in plants lacking *Ph1* than had been previously observed in plants grown in a glasshouse or under controlled environmental conditions in Norwich UK. Some Córdoba meiocytes exhibited up to 21 chiasmata at metaphase I in the absence of *Ph1*, close to that expected from the number of MLH1 sites observed on synapsed homoeologues at diplotene (Martín et al. 2014). We, therefore, tried to replicate this observation in Norwich, in order to determine which of the different Córdoba growth conditions were responsible for the increased level of chiasmata in wheat-rye hybrids lacking *Ph1*. It is widely accepted in wheat studies that each chiasma represents a CO (Fu and Sears 1973); therefore, we use both terms synonymously. We first investigated whether the modified Hoagland solution or different temperature could be responsible for the increased CO number. We performed the experiments both in a glasshouse and under controlled environmental conditions, with similar results; however, only data generated under controlled environmental conditions are presented here.

**Fig. 3:**
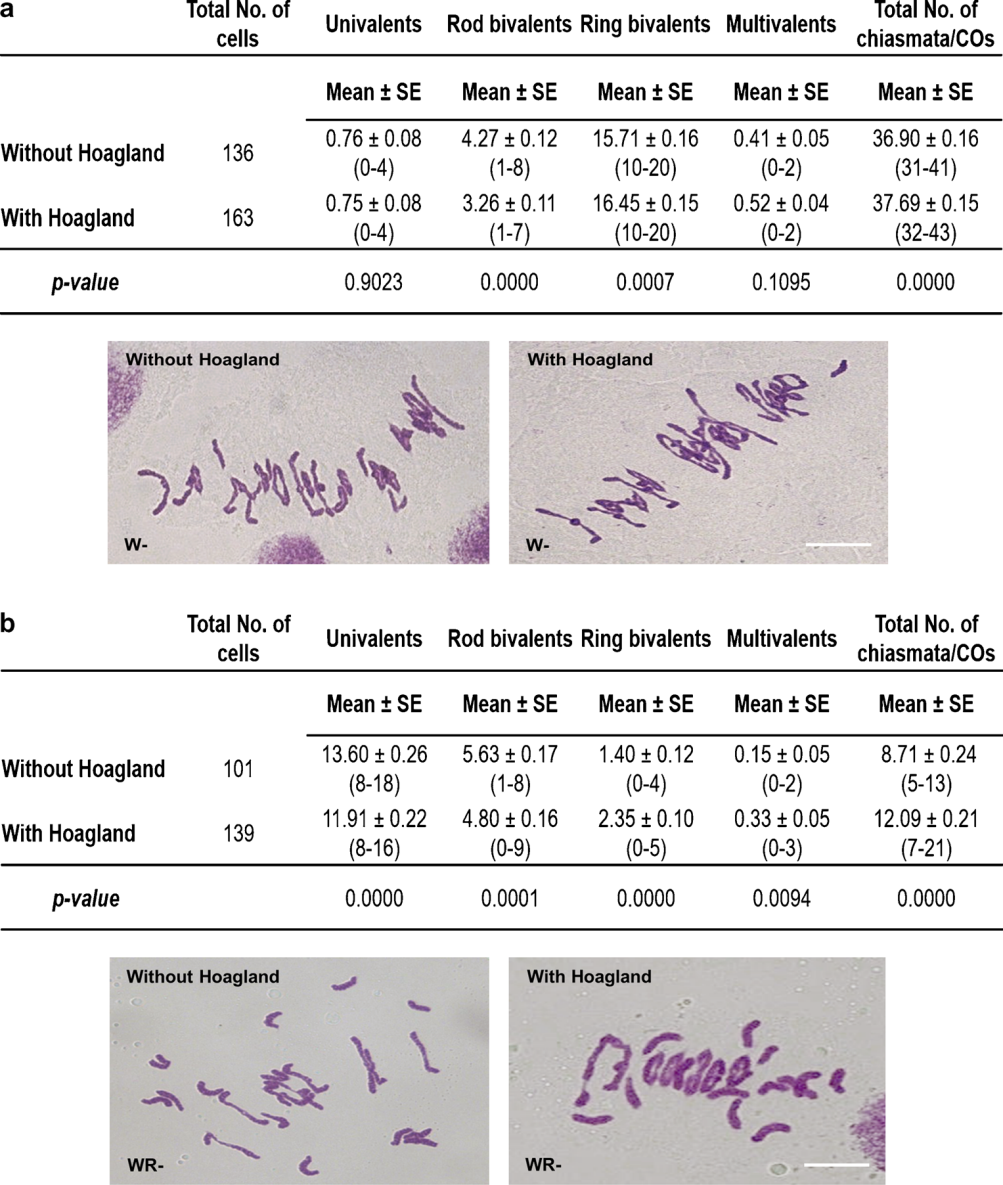
Results of the nutrient solution treatment in wheat and wheat-rye hybrids, in both the presence and absence of the *Ph1* locus. The number of univalents, bivalents, multivalent and total COs was scored at meiotic metaphase I, with and without the addition of a modified Hoagland solution. **a** In wheat in the absence of *Ph1* (W−), the number of ring bivalents and the total number of COs were significantly higher when the nutrient solution was added. **b** In wheat-rye hybrids in the absence of *Ph1* (WR−), the number of ring bivalents and multivalents and the total number of COs were significantly higher in the presence of the nutrient solution. Thus, the presence of the nutrient solution in the soil affects homologous and homoeologous CO frequency in the absence of *Ph1*. *Values in parenthesis* indicate range of variation between cells. *P* < 0.05 indicates significant differences according to LSD test. *Scale bars* represent 10 μm

To assess the effect of nutrient concentration in the soil on homologous and homoeologous CO frequency in meiotic metaphase I, we added a modified Hoagland solution to the soil in which wheat and wheat-rye hybrids, both lacking *Ph1*, were growing at 20 °C. Under these controlled environmental conditions, the modified Hoagland solution had little effect on vegetative growth. However, the treatment significantly increased the number of ring bivalents per meiocyte in wheat lacking *Ph1* (a mean of 16.45 against the 15.71 without Hoagland), with a corresponding reduction in rod bivalents (3.26 with Hoagland and 4.27 without Hoagland) (Fig. 3). GISH labelling of the different wheat genomes (A, B and D) revealed that most ring bivalents were formed between homologues (Online Resource 5), suggesting an increase in homologous CO frequency. The treatment also increased homoeologue CO frequency in the wheat-rye hybrids lacking *Ph1*, shown by an increased number of ring bivalents (2.35 with Hoagland and 1.40 without Hoagland) (Fig. 3b). The number of COs increased from a mean of 8.71 (without Hoagland) to 12.09 (with Hoagland) COs per meiocyte, with up to 21 COs scored in some meiocytes. As described previously (Martín et al. 2014), this is close in number to the 21 MLH1 sites observed on the synapsed homoeologues. Thus, both homoeologous and homologous COs can be increased in wheat lacking *Ph1* by altering the nutrient composition of the soil in which they are grown. Previous studies have suggested that soil nutrient composition in which plants are grown can affect CO formation (Grant 1952; Law 1963; Bennett and Rees 1970; Fedak 1973; Deniz and Tufan 1998). We therefore also treated wheat-rye hybrids carrying *Ph1* with the same nutrient solution; however, no significant increase in CO number was observed (Online Resource 6).

### The level of CO formation in wheat and wheat-rye hybrids in the absence of *Ph1* is affected by temperature

The addition of the Hoagland solution was thus identified as one of the possible causes for the increase in CO number observed in Cordoba, in wheat-rye hybrids, in the absence of *Ph1*. We then decided to also assess the possible effect of temperature. Wheat and wheat-rye hybrids in the absence of *Ph1* were grown under controlled environmental conditions, at either 13 or 30 °C during the meiotic period, without the addition of modified Hoagland solution. Both temperature treatments had little effect on vegetative growth.

**Fig. 4:**
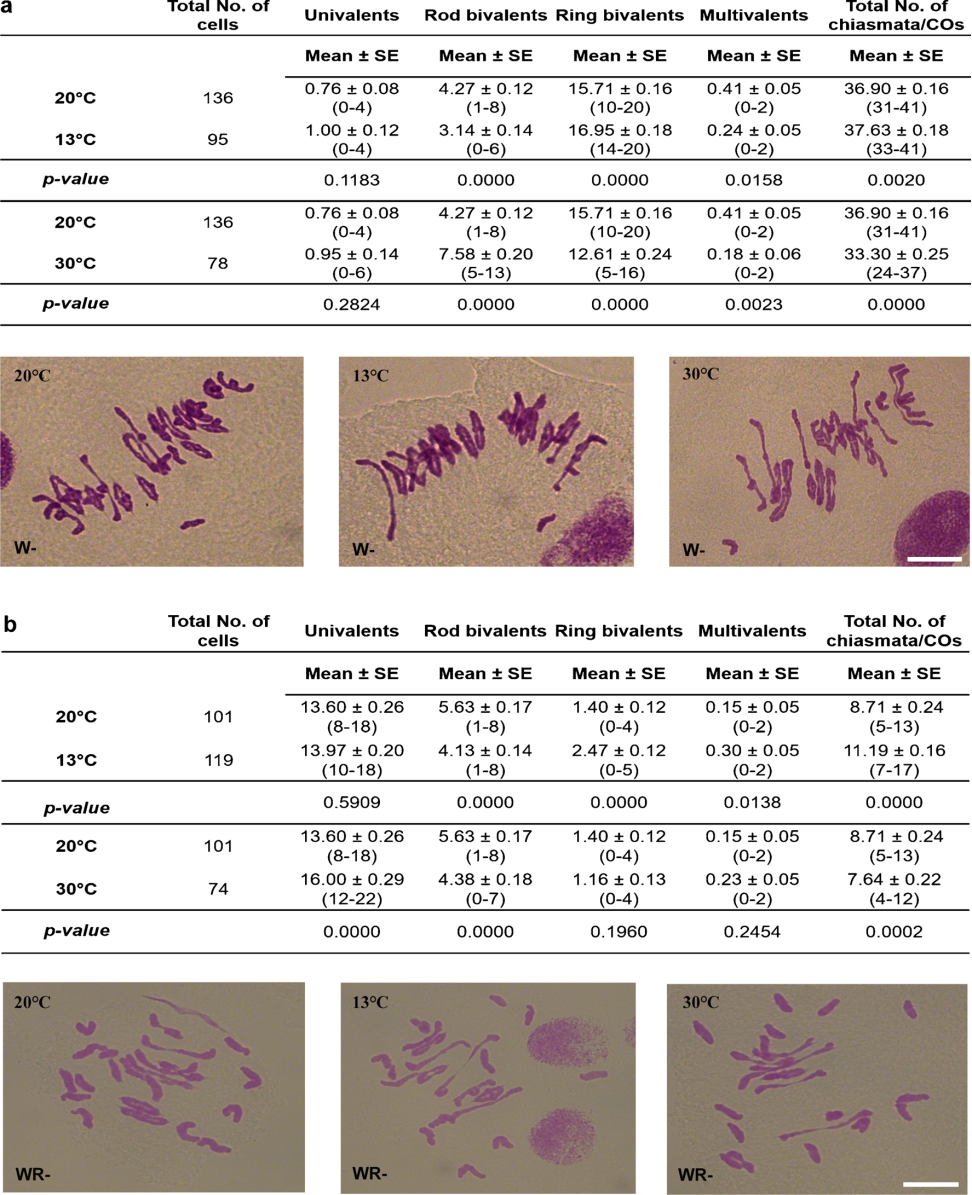
Results of the temperature treatments in wheat and wheat-rye hybrids, both in the absence of the *Ph1* locus. The number of univalents, bivalents, multivalents and total COs was scored at meiotic metaphase I at 20 °C (controls), 13 °C and 30 °C. **a** In wheat in the absence of *Ph1* (W−), the number of ring bivalents and the total number of COs were significantly higher when plants were grown at 13 °C and significantly lower when plants were grown at 30 °C. **b** In wheat-rye hybrids in the absence of *Ph1* (WR−), the number of ring bivalents and multivalents and the total number of COs were significantly higher at 13 °C. However, the number of ring bivalents and the total number of COs were lower at 30 °C. Thus, both low and high temperature affect homologous and homoeologous CO frequency in the absence of *Ph1*. *Values in parenthesis* indicate range of variation between cells. *P* < 0.05 indicates significant differences according to LSD test. *Scale bars* represent 10 μm

At 13 °C, the total number of COs was significantly increased in both the wheat (Fig. 4a) and the wheat-rye hybrids (Fig. 4b), in the absence of *Ph1*. The number of COs increased in wheat from a mean of 36.90 at 20 °C to a mean of 37.63 at 13 °C and in wheat-rye hybrids from a mean of 8.71 at 20 °C to a mean of 11.19 at 13 °C. Both genotypes showed an increase in the number of ring bivalents with a corresponding reduction in rod bivalents (Fig. 4).

Conversely, at 30 °C, the total number of COs was significantly decreased in both the wheat (Fig. 4a) and the wheat-rye hybrids (Fig. 4b), in the absence of *Ph1*. The number of COs decreased in wheat from a mean of 36.90 at 20 °C to a mean of 33.30 at 30 °C and in wheat-rye hybrids from a mean of 8.71 at 20 °C to a mean of 7.64 at 30 °C. The lowering of chiasma frequency at high temperature was accompanied by an increased frequency of univalents and rod bivalents in wheat and an increase of univalents in wheat-rye hybrids, both in the absence of *Ph1*. Thus, in the absence of *Ph1*, the temperature at which wheat is grown can affect the frequency of CO, being significantly increased at low temperature (13 °C) and decreased at high temperature (30 °C). These results are consistent with previous studies in wheat, maize, Arabidopsis and barley, reporting that growth temperature can influence the formation of COs during meiosis (Bayliss and Riley 1972; Verde 2003; Francis et al. 2007; Higgins et al. 2012). In contrast, wheat-rye hybrids carrying *Ph1* grown at either 20 or 13 °C exhibited 0.59 and 0.77 COs at metaphase I respectively. This level of CO is not significantly great to exploit practically. We did not study the effect of high temperatures on CO frequency in wheat (or wheat-rye hybrids) carrying *Ph1*, as it has already been reported that such temperatures reduce CO frequency (Bayliss and Riley 1972).

Overall, these results suggest that, in the absence of *Ph1*, temperature and soil nutrient composition can affect the level of homologous and homoeologous COs in wheat and wheat hybrids. In the wheat-rye hybrids, both treatments lead to an increase in conversion of rod bivalents to ring bivalents at metaphase I. This suggests an increase in COs between synapsed homoeologues, rather than an increased association of ectopic sites. Genetic map analysis of wheat in the presence and absence of *Ph1* showed a similar distribution of COs along chromosomes (Dubcovsky et al. 1995), suggesting that the *Ph1* locus affects the level of MLH1-dependent class I interfering COs, but not their distribution. Thus, it is unlikely that nutrients and/or temperature treatments lead to an altered distribution of COs along synapsed chromosomes in the absence of *Ph1*. It is more likely that temperature and one or more components of the modified Hoagland solution affect the MLH1 complex activity on the double Holliday junction or affect the double Holliday junction conformation itself, allowing the junction to resolve as a CO rather than a non-CO. For instance, changes in magnesium concentration have been shown to affect the conformation of double Holliday junctions, which may then influence CO frequency (Yu et al. 2004; Ranjha et al. 2014).

### The *Ph1* is a complex locus: proposal for the mode of action of the *Ph1* locus

*Ph1* is a complex locus, and after almost 60 years since its discovery, we still do not know its exact mode of action. Molecular characterisation of the *Ph1* locus combined cereal synteny and wheat BAC contiging, with metaphase I analysis of mutants carrying deletions of chromosome 5B (Roberts et al. 1999; Griffiths et al. 2006; Al-Kaff et al. 2008). Finally, it was defined to a region containing a cluster of Cdk2-like and S-adenosyl methionine-dependent methyltransferase (SAM-MTases) genes, with a duplicated segment of heterochromatin from 3B inserted into this cluster. This heterochromatin segment also contains a gene originally designated as hypothetical 3 (Hyp3) (Griffiths et al. 2006; Al-Kaff et al. 2008), which has been now reannotated as ZIP4 (UniProtKB—Q2L3T5).

In terms of the role of *Ph1* during meiosis, we now know that *Ph1* stabilises polyploidy in wheat through two different mechanisms: by controlling the accuracy of homologous synapsis during early meiosis and by regulating CO formation later in meiosis (Martín et al. 2014). We propose that the effect of *Ph1* on synapsis is probably a consequence of a change in chromatin structure produced by the Cdk-like and SAM-MTase cluster. The involvement of Cdk2 in H1 phosphorylation, replication, chromatin condensation and synapsis between non-homologous chromosomes has been widely reported (Krasinska et al. 2008; Viera et al. 2009). We have already shown that the levels of histone H1 phosphorylation, pre-meiotic replication and chromatin structure are all altered by *Ph1* (Greer et al. 2012), which may affect chromatin structure and hence the efficiency of synapsis. On the other hand, although ZIP4 has been reported to affect both synapsis and CO formation in yeast (Tsubouchi et al. 2006), the effect in plants is mainly on CO formation (Chelysheva et al. 2007; Shen et al. 2012). Thus, it seems more likely that ZIP4 is involved in the effect of *Ph1* on CO formation. However, there are still no data to elucidate how ZIP4 contributes to the effect of *Ph1* on the CO process. This question cannot be answered using ZIP4 RNAi-based approaches because this would result in overall ZIP4 activity suppression, leading to sterility (Chelysheva et al. 2007; Shen et al. 2012). However, the question will most likely be answered in the near future, using other approaches such as analysis of the recently available tilling mutants (www.wheat-training.com/tilling-mutant-resources) and other transformation approaches such as the CRISPR genome editing technique.

## Conclusion

The *Ph1* locus was assumed to prevent pairing and synapsis between homoeologues or related chromosomes; however, it has recently been reported that *Ph1* does not prevent homoeologous synapsis (Martín et al. 2014). In this study, we show that wheat possesses a basic mechanism for sorting homologues from homoeologues during the telomere bouquet stage, independent of *Ph1*. During this telomere bouquet stage, only homologous synapsis occurs, while homoeologous synapsis starts later during telomere bouquet dispersal. In hexaploid wheat, the *Ph1* locus promotes early homologous synapsis, thus avoiding homoeologous synapsis. In the absence of *Ph1*, homologous synapsis is delayed, allowing a degree of homoeologous synapsis to also take place in up to 8 of the 42 chromosomes.

The *Ph1* locus has also been reported to have a major effect on homoeologous CO formation, by preventing MLH1 sites on synapsed homoeologues from becoming COs. Although the absence of *Ph1* allows some of these MLH1 sites to become COs, still a significant proportion of these sites are processed into non-COs. In this study, we reveal that the level of MLH1 sites progressing to COs can be increased in the absence of *Ph1* by altering selected environmental factors, such as nutrient composition in the soil and temperature. We show that increased nutrients and lower temperature can increase the level of CO. These observations highlight the importance of growth conditions on wheat meiosis, which may allow improved exploitation of the *Ph1* locus in breeding programmes.

## Electronic supplementary material

ESM 1 (DOCX 2517 kb)
