# Supplementary material for: Dual effect of the wheat Ph1 locus on chromosome synapsis and crossover
Source: Chromosoma. 2017 Apr 1;126(6):669–80. doi: 10.1007/s00412-017-0630-0 (PMC5688220; doi:10.1007/s00412-017-0630-0)
Supplement: Supplementary file 1 — (DOCX 2517 kb) [file 412_2017_630_MOESM1_ESM.docx]

**Electronic Supplementary Material**

**“Dual effect of the wheat *Ph1* locus on chromosome synapsis and crossover”**

**Chromosoma journal**

Azahara C. Martín^1^, María-Dolores Rey^1^, Peter Shaw, Graham Moore

^1^ Joint first author

**Author for correspondence:**

Professor Graham Moore

John Innes Centre, Norwich Research Park, Norwich NR4 7UH, UK

Tel:       +44 (0)1603 450577
[graham.moore@jic.ac.uk](mailto:graham.moore@jic.ac.uk)

**Online Resource 1** Homoeologous synapsis in wheat-rye meiocytes in the presence (WR+) and absence (WR-) of *Ph1* during the telomere bouquet.

Immunolocalisation of meiotic proteins ASY1 (green) and ZYP1 (magenta), combined with telomeres (magenta) labelled by FISH. Short tracks of ZYP1 labelling are detected in some WR+ and WR- meiocytes during the telomere bouquet stage (group 1). 87% of the WR+ meiocytes showed small tracks of ZYP1 being loaded, while only 17% of the WR- showed the short tracks. This suggests that the absence of *Ph1* slightly delays synapsis of homoeologues in wheat-rye hybrids during the telomere bouquet stage. DAPI staining in blue. *Scale bar* represents 10 µm.


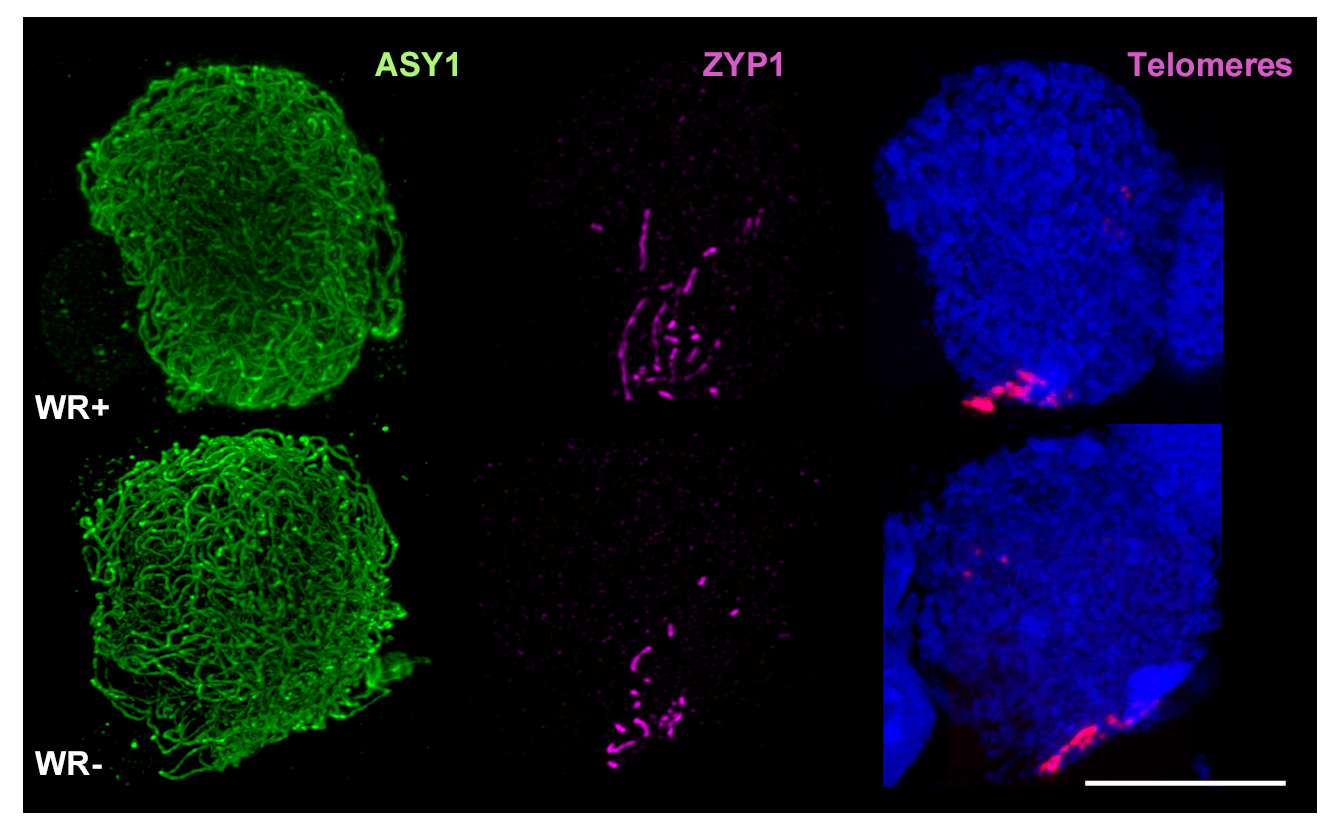


**Online Resource 2** Homoeologous synapsis in wheat-rye meiocytes in the presence (WR+) and absence (WR-) of *Ph1* after telomere bouquet dispersal.

Immunolocalisation of meiotic proteins ASY1 (green) and ZYP1 (magenta), combined with telomeres (magenta) labelled by FISH. Although almost no ZYP1 labelling was detected in wheat-rye meiocytes during the telomere bouquet, long tracks of ZYP1 labelling are observed in both WR+ and WR- after telomere bouquet dispersal, reaching the same levels of synapsis in both WR+ and WR-. This indicates that homoeologous synapsis takes place mostly after the telomere bouquet. DAPI staining in blue. *Scale bar* represents 10 µm.


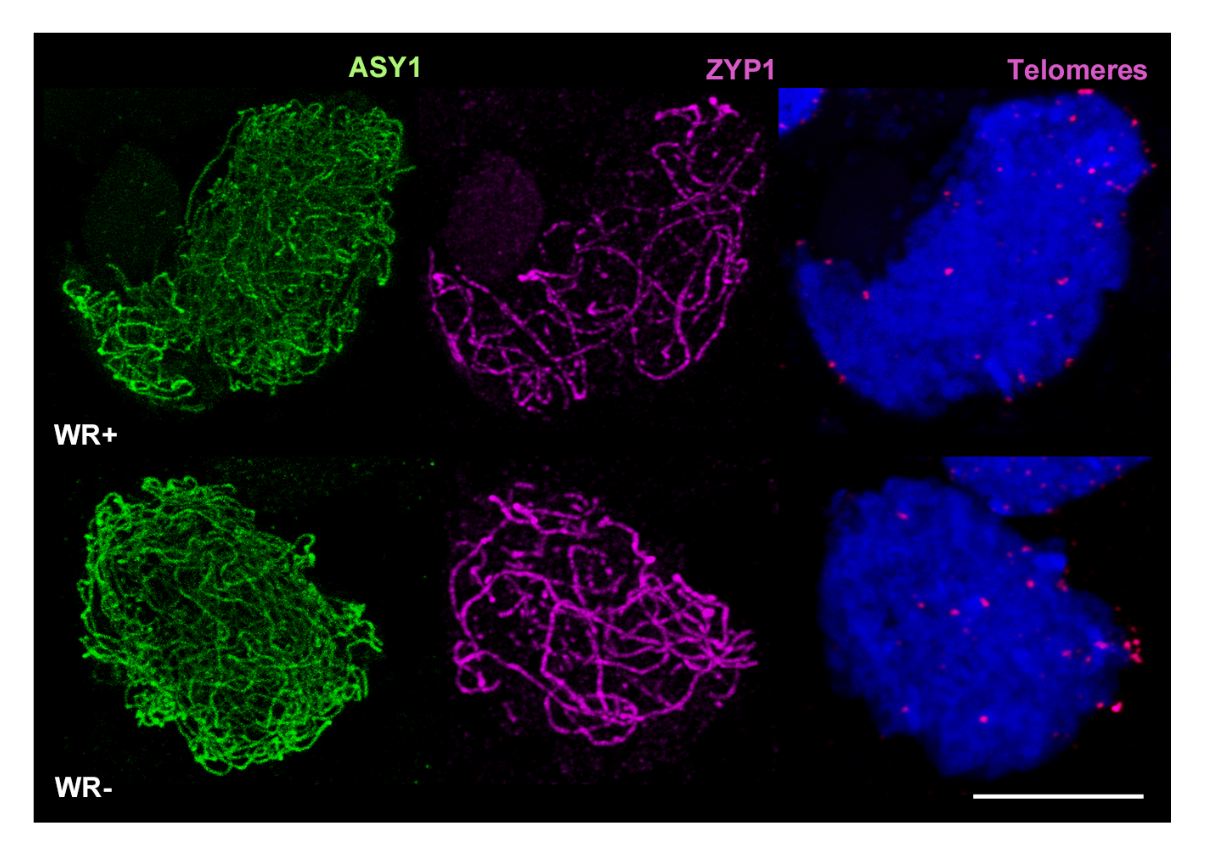


**Online Resource 3** Synapsis in wheat meiocytes in the presence (W+) and absence (W-) of *Ph1* during pachytene.

Immunolocalisation of meiotic proteins ASY1 (green) and ZYP1 (magenta), combined with telomeres (magenta) labelled by FISH. Only ZYP1 and no ASY1 is observed in W+ and W-, indicating that synapsis is completed in wheat, both in the presence and absence of the *Ph1* locus. *Scale bar* represents 10 µm.


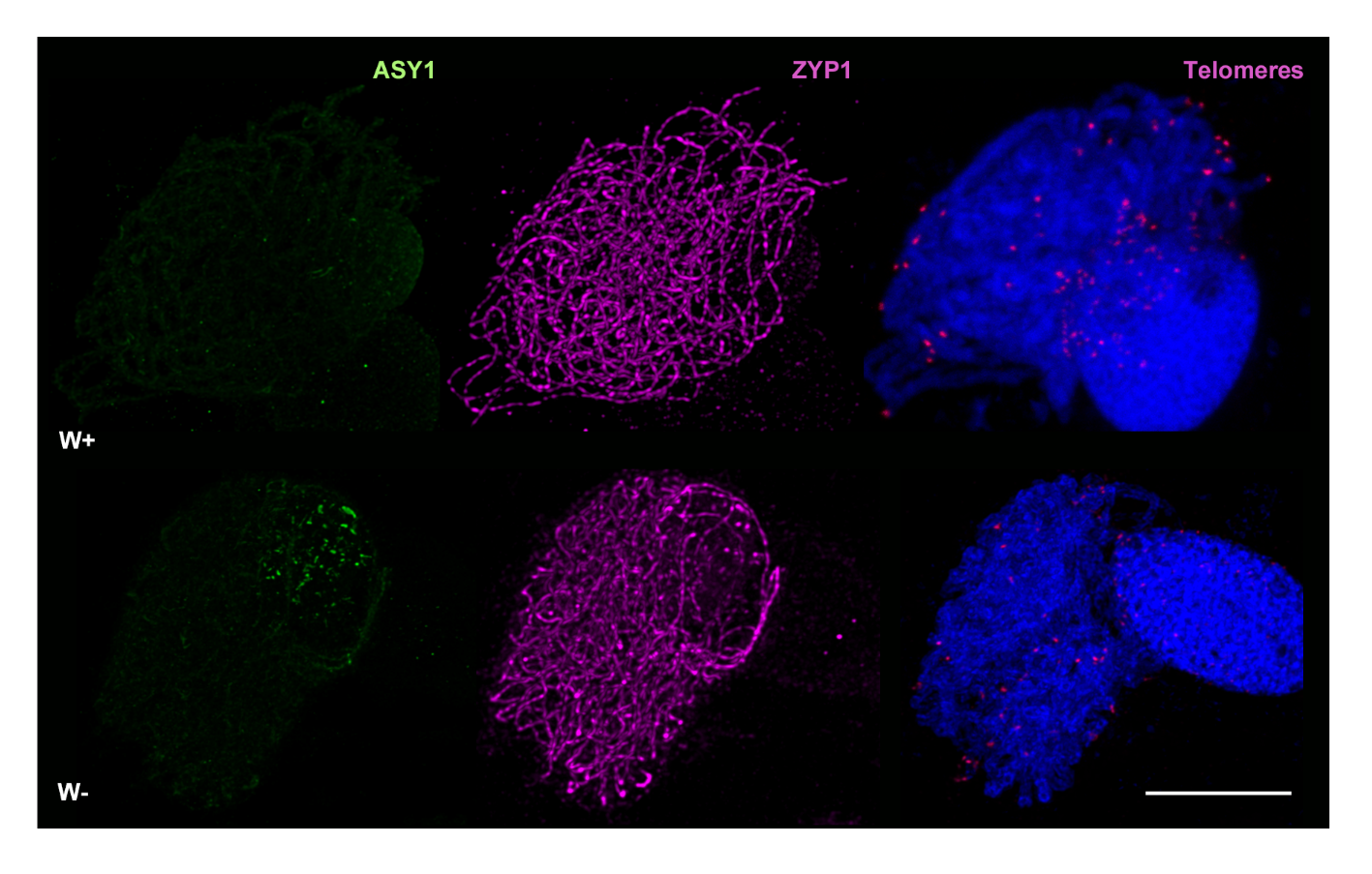


**Online Resource 4** Number of 4P6 signals observed during telomere bouquet dispersal (group 2) in wheat in the presence (W+) and absence (W-) of *Ph1*.

In the presence of *Ph1*, 85.7% of the meiocytes displayed 7 to 10 signals. In the absence of *Ph1* only 25% of the meiocytes showed 7 to 10 signal. This result indicates that chromosome pairing is delayed during telomere bouquet dispersal in the absence of *Ph1*. *Scale bar* represents 10 µm.

| **Genotype** | **Number of 4P6 signals** | **Number of cells** | **Genotype** | **Number of 4P6 signals** | **Number of cells** |
| --- | --- | --- | --- | --- | --- |
| **W+** | 7 to 10 | 42 (85.7 %) | **W-** | 7 to 10 | 14 (25 %) |
|  | > 10 | 7 (14.3 %) |  | > 10 | 42 (75 %) |

**Online Resource 5** Genomic *in situ* hybridisation (GISH) of meiotic metaphase I chromosomes in wheat lacking *Ph1* after treatment with a modified Hoagland solution*.*

Genomic DNA of *Triticum urartu* (magenta), *Aegilops speltoides* (grey) and *Aegilops tauschii* (green) were used as probes to label the A, B, and D genome respectively. After addition of the nutrient solution, most ring bivalents were formed between homologues in wheat lacking *Ph1*. *Scale bar* represents 10 µm.


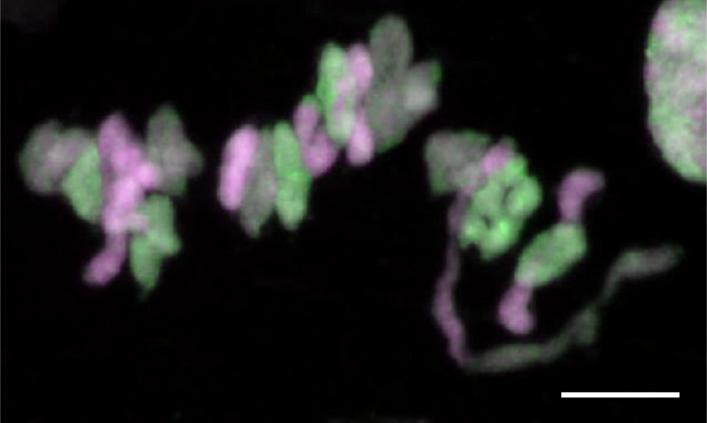


**Online Resource 6** Results of the nutrient solution treatment in wheat-rye hybrids in the presence of the *Ph1* locus.

The number of univalents, bivalents and COs were scored at metaphase I, with and without the addition of a modified Hoagland solution. As shown in the Table, addition of the Hoagland solution to the soil does not affect homologous and homoeologous CO frequency in presence of *Ph1*. Values are given as mean and standard error of the mean (mean ± SD). Values in parenthesis indicate range of variation between cells. *P > 0.05* indicates no significant differences.

|  | **Total No. of cells** | **Univalents** | **Rod bivalents** | **Total No. of chiasmata/COs** |
| --- | --- | --- | --- | --- |
| Without Hoagland | 80 | **Mean ± SE** | **Mean ± SE** | **Mean ± SE** |
|  |  | 26.83 ± 0.22  (22-28) | 0.59 ± 0.11  (0-3) | 0.59 ± 0.11  (0-3) |
| With Hoagland | 118 | 26.29 ± 0.18  (20-28) | 0.85 ± 0.10  (0-4) | 0.85 ± 0.10  (0-4) |
|  | ***p*-value** | 0.3448 | 0.1325 | 0.1325 |
